# Machine learning-based predictive model for enteral nutrition-associated diarrhea in ICU patients and its nursing applications

**DOI:** 10.3389/fnut.2025.1584717

**Published:** 2025-06-25

**Authors:** Xiaoying Liao, Chunhua Li, Qunyan Liu, Wang Xia, Zhenglin Liu, Jiamao Zhu, Wei Hu, Qionghua Hong

**Affiliations:** ^1^Shangrao People's Hospital, Shangrao, China; ^2^School of Nursing, Jinzhou Medical University, Jinzhou, China

**Keywords:** enteral nutrition-associated diarrhea, machine learning, random forest, feature importance, critically ill patients

## Abstract

**Background:**

Enteral Nutrition-Associated Diarrhea (ENAD) is a common complication in critically ill patients, significantly impacting clinical outcomes. Accurately predicting the risk of ENAD is crucial for early intervention and improving patient care.

**Objective:**

This study aims to develop and validate a machine learning (ML)-based risk prediction model for Enteral Nutrition-Associated Diarrhea (ENAD) in ICU patients, and explore its application in nursing practice.

**Method:**

This study was conducted from January 2023 to October 2024 in the Comprehensive Intensive Care Unit (ICU) of a tertiary hospital in China, retrospectively analyzing data from ICU patients receiving enteral nutrition. LASSO regression was used for feature selection, and 9 machine learning (ML) algorithms were evaluated. Model performance was assessed using metrics such as the area under the receiver operating characteristic curve (AUC). The SHapley Additive exPlanation (SHAP) method was employed to interpret feature importance and determine the final model.

**Results:**

Among the 9 ML models, the random forest (RF) model demonstrated the highest discriminative ability, achieving an AUC (95% CI) of 0.777 (0.702–0.830). After dimensionality reduction based on feature importance analysis, a simplified and interpretable RF model with 12 key predictors was established, yielding an AUC (95% CI) of 0.754 (0.685–0.823).

**Conclusion:**

The RF-based predictive model developed in this study provides a reliable and interpretable tool for identifying the risk of ENAD in ICU patients, contributing to targeted nursing interventions and improved patient outcomes. The research highlights the potential of machine learning in enhancing clinical decision-making and personalized care.

## Introduction

Enteral nutrition (EN) is a vital supportive therapy for critically ill patients in intensive care units (ICUs), providing essential nutritional support and enhancing clinical outcomes (1, 2). However, enteral nutrition-associated diarrhea (ENAD) remains a significant clinical challenge, affecting approximately 20–30% of critically ill patients and contributing to increased morbidity, extended hospital stays, and substantial healthcare costs (3–5).

The pathogenesis of ENAD is multifactorial, involving complex interactions between nutritional formulations, patient physiological status, medications, and underlying medical conditions (6–8). Current evidence identifies several key categories of ENAD risk factors that require comprehensive assessment and monitoring. Patient-specific factors include advanced age, severity of illness (as measured by APACHE II or SOFA scores), pre-existing gastrointestinal disorders, hypoalbuminemia, electrolyte imbalances (particularly hyponatremia), and compromised immune status (9). Nutritional formulation-related factors encompass formula osmolality, fiber content, protein concentration, fat composition, and the use of specialized formulations such as elemental or semi-elemental products. Mixed feeding regimens, involving concurrent administration of different formula types, have been associated with increased gastrointestinal intolerance and diarrheal episodes (10). Feeding delivery methods significantly influence ENAD development, with continuous versus bolus feeding patterns, feeding rate progression, gastric residual volume management, and feeding tube positioning all contributing to risk stratification (11). Medication-related factors include concurrent antibiotic therapy (particularly broad-spectrum agents), proton pump inhibitors, prokinetic agents, and medications affecting gastrointestinal motility (12). Environmental and care-related factors such as ICU temperature control, stress levels, and nursing care protocols also contribute to ENAD risk. Traditional risk assessment methods have been limited by single-dimensional evaluation approaches, making them insufficient for capturing the complex and dynamic nature of nutritional disorders. Emerging precision medicine strategies are increasingly developing comprehensive multidimensional assessment models (13). These include machine learning-based predictive algorithms, integrated assessments of clinical, biomarker, and genomic information, and intelligent systems for dynamic real-time nutritional risk monitoring. These innovative methodologies utilize advanced algorithmic models to integrate extensive clinical datasets, enabling rapid multidimensional information analysis, precise identification of high-risk populations, personalized risk stratification, and early warning interventions with enhanced accuracy in managing nutritional disorders (14).

Machine learning (ML) has emerged as a powerful approach in clinical prediction, offering superior capabilities in handling complex, non-linear relationships within medical data (15, 16, 44). Recent advances in ML algorithms have demonstrated remarkable potential in developing predictive models across various medical domains, including critical care, by leveraging advanced feature selection and interpretation techniques (17, 18).

Despite the promising potential of machine learning in clinical risk prediction, significant research gaps persist in developing comprehensive risk assessment models for ENAD. Current studies are predominantly constrained by limited sample sizes (typically <200 cases), narrow feature selection (primarily focusing on basic demographic indicators), and insufficient model interpretability (19). These limitations critically impede the widespread clinical implementation of predictive models. Moreover, traditional machine learning approaches function as “black box” systems, lacking transparency in key risk-driving factors and consequently undermining clinicians’ confidence in model-derived decisions (20). Therefore, there is an urgent need to develop more comprehensive, interpretable, and high-precision ENAD risk prediction models. By integrating multidimensional clinical data, such models could ultimately enhance the accuracy and clinical utility of risk assessment strategies (21).

## Methods

### Study population

This study retrospectively enrolled 756 critically ill patients who received enteral nutrition (EN) support in the General Intensive Care Unit (ICU) of Shangrao People’s Hospital between January 2023 and October 2024. The sample size was calculated by integrating machine learning model complexity with clinical requirements, following the modified event-to-feature ratio (EPV) criterion proposed by Vabalas et al. (45), which recommends EPV ≥ 15 for nonlinear models (22). To address potential overfitting risks associated with the observed EPV of 8.2 (189 events/24 features), rigorous mitigation strategies—including nested cross-validation (5 outer folds and 3 inner folds) and regularization techniques—were implemented. Data were systematically extracted from electronic medical records, ICU nursing documentation, and laboratory databases, followed by feature engineering to construct a structured dataset encompassing 24 predictive variables across five domains: demographics (age, sex), disease severity (diagnosis category, APACHE II score, mechanical ventilation duration), therapeutic interventions (vasopressor use, sedation-analgesia protocols, antibiotic duration), biomarkers (albumin, C-reactive protein, electrolytes), and EN parameters (formula type, infusion rate, heater use). Inclusion criteria required: (1) adults (≥18 years) requiring mechanical ventilation for >24 h; (2) standardized EN administration ≥48 h (compliant with ESPEN guidelines); (3) APACHE II score ≥15 at ICU admission; and (4) complete documentation of all 24 study variables, including time-sensitive EN metrics. Exclusion criteria comprised: (1) chronic gastrointestinal diseases or recent gastrointestinal surgery (≤30 days); (2) EN interruption <24 h or critical variable missingness >20%; (3) end-stage conditions (life expectancy <72 h); or (4) concurrent participation in other nutritional intervention trials. Analysis of comorbidities and diarrhea events did not show any statistically significant association (χ^2^ = 11.01; *p* = 0.357). Malignancies (38.1%) were associated with the highest diarrhea incidence; next: cardiovascular/cerebrovascular (34.6%) and patients without comorbidities 32.8%; the lowest for respiratory and gastrointestinal co-morbidities (21.4%) Adherence to multiple comorbidity groups showed a high prevalence of diarrhea, even in the absence of concomitant illness which implies that diarrhea development may not be related exclusively with underlying conditions. Supplementary Figure 1: the incidence of diarrhea based on comorbidity groups in the study population.

### Data collection and processing

**Figure 1:**
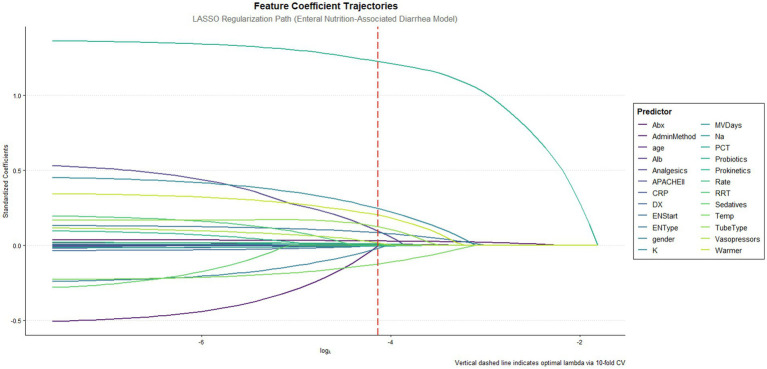
Feature coefficient trajectories along the lasso regularization path. This figure illustrates the feature coefficient trajectories of the LASSO regression model fitted to predict enteral nutrition-associated diarrhea. The x-axis shows the log of the regularization parameter (log *λ*), while the y-axis represents the standardized coefficients of the predictive features. The vertical red dashed line indicates the optimal λ determined by 10-fold cross-validation.

This study developed predictive models utilizing multi-dimensional data collected within 24 h of ICU admission, incorporating 24 initial variables across five domains: demographic characteristics (gender, age), clinical features (disease categories, APACHE II scores, mechanical ventilation days), therapeutic interventions (renal replacement therapy, antibiotic days, prokinetics/probiotics/ vasopressors/analgesics/sedatives administration), enteral nutrition parameters (formula types, infusion rate, tube type, warmer usage, initiation time), and laboratory indices (albumin, electrolytes, inflammatory biomarkers). Using R’s caret package, 756 patients were stratified by diarrhea outcomes and partitioned into a 70% training set (*n* = 529) and a 30% test set (*n* = 227), with the latter stringently isolated from the feature selection processes to prevent data leakage. LASSO regression (10-fold cross-validation, *λ* = “lambda.1se”) conducted exclusively in the training set identified 18 non-redundant predictors (Figure 1), including age, APACHE II scores, antibiotic duration, probiotics use, serum sodium levels, and enteral nutrition-related procedural parameters. Continuous variables were expressed as medians with interquartile ranges (IQR) and analyzed via Mann–Whitney U tests, while categorical variables were presented as frequencies (percentages) with χ^2^ tests. Statistical significance was defined as two-tailed *p* < 0.05, with precise *p*-values reported unless below 0.001 (Table 1).

**Table 1:** Demographic and clinical characteristics of critical care patients nondiarrhea and diarrhea.

Characteristics	Subgroups	All patients (*n* = 756)	Nondiarrhea (*n* = 539)	Diarrhea (*n* = 217)	*p* value*
Gender	Male	531 (70.2)	371 (68.8)	160 (73.7)	0.213
	Female	225 (29.8)	168 (31.2)	57 (26.3)	
DX	Infectious diseases	241 (31.9)	167 (31.0)	74 (34.1)	0.750
	Trauma	155 (20.5)	113 (21.0)	42 (19.4)	
	Acute organ failure	207 (27.4)	146 (27.1)	61 (28.1)	
	Metabolic/toxic/special	49 (6.5)	34 (6.3)	15 (6.9)	
	Tumor	104 (13.8)	79 (14.7)	25 (11.5)	
RRT	No	543 (71.8)	401 (74.4)	142 (65.4)	0.017
	Yes	213 (28.2)	138 (25.6)	75 (34.6)	
Prokinetics	No	499 (66.0)	379 (70.3)	120 (55.3)	<0.001
	Yes	257 (34.0)	160 (29.7)	97 (44.7)	
Probiotics	No	414 (54.8)	363 (67.3)	51 (23.5)	<0.001
	Yes	342 (45.2)	176 (32.7)	166 (76.5)	
Vasopressors	No	172 (22.8)	133 (24.7)	39 (18.0)	0.058
	Yes	584 (77.2)	406 (75.3)	178 (82.0)	
Analgesics	No	100 (13.2)	78 (14.5)	22 (10.1)	0.141
	Yes	656 (86.8)	461 (85.5)	195 (89.9)	
Sedatives	No	108 (14.3)	88 (16.3)	20 (9.2)	0.016
	Yes	648 (85.7)	451 (83.7)	197 (90.8)	
EN Type	TPF	158 (20.9)	121 (22.4)	37 (17.1)	<0.001
	SP	224 (29.6)	176 (32.7)	48 (22.1)	
	Peptison	76 (10.1)	61 (11.3)	15 (6.9)	
	Fresubin	58 (7.7)	44 (8.2)	14 (6.5)	
	Mixed feeding	240 (31.7)	137 (25.4)	103 (47.5)	
Admin method	EN specific pump	29 (3.8)	21 (3.9)	8 (3.7)	0.893
	Non-specific pump	714 (94.4)	508 (94.2)	206 (94.9)	
	Syringe push	13 (1.7)	10 (1.9)	3 (1.4)	
Tube type	Gastric tube	656 (86.8)	482 (89.4)	174 (80.2)	0.001
	Nasoenteral tube	100 (13.2)	57 (10.6)	43 (19.8)	
Warmer	N	415 (54.9)	314 (58.3)	101 (46.5)	0.004
	Yes	341 (45.1)	225 (41.7)	116 (53.5)	
Age (years), M (IQR)		68.0 (57.0–76.0)	67.0 (56.5–75.0)	71.0 (60.0–78.0)	0.003
APACHE II score, M (IQR)		25.0 (19.0–29.0)	25.0 (19.0–29.0)	25.0 (20.0–28.0)	0.406
MVDays (days), M (IQR)		5.0 (1.2–10.2)	4.0 (1.0–8.9)	7.0 (2.0–14.8)	<0.001
Abx (days), M (IQR)		12.0 (7.0–19.0)	10.0 (6.0–16.0)	16.0 (11.0–26.0)	<0.001
Alb (g/L), M (IQR)		32.2 (28.8–36.1)	32.1 (28.8–36.2)	32.4 (28.8–35.9)	0.895
K (mmol/L), M (IQR)		3.9 (3.5–4.3)	3.9 (3.5–4.3)	4.0 (3.5–4.5)	0.065
Na (mmol/L), M (IQR)		139.0 (136.0–143.7)	139.0 (135.2–143.0)	140.0 (136.0–144.1)	0.048
CRP (mg/L), M (IQR)		62.4 (26.6–119.2)	68.2 (27.0–123.0)	55.7 (26.0–105.9)	0.152
PCT (ng/mL), M (IQR)		0.8 (0.2–3.4)	0.8 (0.2–3.7)	0.8 (0.2–2.9)	0.842
Rate (mL/h), M (IQR)		50.0 (45.0–55.0)	50.0 (45.0–55.0)	50.0 (45.0–55.0)	0.05
Temp (°C), M (IQR)		21.0 (20.0–22.0)	21.0 (20.0–22.0)	20.0 (20.0–22.0)	<0.001
ENStart (days), M (IQR)		3.0 (2.0–5.0)	3.0 (2.0–5.0)	2.0 (1.0–5.0)	0.336

Abbreviations used in this study included: APACHE II, Acute Physiology and Chronic Health Evaluation II, range 0–71; RRT, renal replacement therapy; EN, enteral nutrition; CRP, C-reactive protein; PCT, procalcitonin. Disease categories were classified as infectious diseases, trauma/injury, acute organ failure, metabolic/toxicological/special conditions, and oncological diseases.

Binary variables (0 = no, 1 = yes) included RRT, prokinetics, probiotics, vasopressors, analgesics, sedatives, warmer use, and the primary outcome of diarrhea. Gender was coded as 1 (male) or 2 (female). EN-related variables included formula types (TPF, SP, whole protein formula, peptide formula, and mixed feeding), administration methods (dedicated EN pump, non-dedicated pump, syringe push), and tube types (gastric or post-pyloric).

Laboratory parameters included CRP, PCT, albumin, potassium, and sodium, all measured within standard ranges. Other continuous variables included age (years), mechanical ventilation days, antibiotic therapy duration (days), EN initiation time (days from ICU admission), body temperature (°C), and EN administration rate (mL/h).

### Definition of diarrhea

The diagnosis of enteral nutrition-associated diarrhea was rigorously defined according to the ASPEN/ESPEN joint working group criteria (23), requiring the concurrent presence of two criteria: (1) abnormal stool consistency classified as Type ≥6 on the Bristol Stool Form Scale (liquid or watery stool), and (2) altered defecation frequency/volume, manifested as ≥3 bowel movements per day or total stool output exceeding 500 g/24 h.

### Operational definitions of nursing-related variables

Mixed Feeding Formulas: Combination of two or more different enteral nutrition products administered within a 24-h period, including concurrent use of standard polymeric formulas with specialized formulations (e.g., elemental, semi-elemental, or disease-specific formulas). This nursing intervention was documented when nurses administered different formula types during the same shift or when feeding regimens were changed more than once daily based on clinical assessment of tolerance or physician orders.

#### Probiotic use

Administration of live microorganisms (including single-strain or multi-strain preparations) via enteral route as documented in nursing medication administration records. This included both prophylactic probiotics ordered for gastrointestinal protection and therapeutic probiotics prescribed for existing digestive complications. Nursing documentation captured the specific probiotic product, dosage, frequency, and duration of administration.

#### Environmental temperature management

Nursing interventions to maintain and monitor ambient room temperature in the ICU patient care environment. This included documentation of room temperature measurements taken during routine nursing assessments (typically every 4–8 h), adjustment of environmental controls (heating/cooling systems), and use of additional warming or cooling devices as nursing interventions to maintain patient comfort and physiological stability.

#### Enteral nutrition feeding rate

The milliliters per hour (mL/h) of enteral formula delivered as documented in nursing feeding administration records. This included both continuous feeding rates (when feeds were administered over 24 h) and calculated hourly rates for intermittent feeding schedules. Nursing assessment of feeding tolerance and rate adjustments based on patient response were documented according to institutional protocols.

#### Antibiotic duration

Total consecutive days of systemic antibiotic therapy as documented in nursing medication administration records from ICU admission to discharge. This included all routes of administration (intravenous, oral, enteral) and was calculated based on actual nursing documentation of medication administration, regardless of changes in specific antibiotic agents during the treatment course.

### Model development and comparison

This study constructed predictive models using 18 variables selected through LASSO regression. Logistic regression was used as the baseline model to evaluate predictive performance, with comparisons made to eight additional machine learning algorithms: support vector machine (SVM), random forest, XGBoost, LightGBM, neural network, AdaBoost, decision tree, and naïve Bayes. The dataset was partitioned into a training set and an independent validation set, with strict isolation of the validation cohort to prevent overfitting. Hyperparameter optimization for all models, including logistic regression, was performed via grid search with 5-fold cross-validation.

Model performance was evaluated comprehensively using area under the ROC curve (AUC), recall, accuracy, F1-score, precision, negative predictive values (NPV), and calibration metrics, with logistic regression serving as the reference model. The validation framework incorporated 5-fold and 10-fold cross-validation iterations within the training cohort, followed by final evaluation on the independent test set. Robust confidence intervals for all metrics were derived through Bootstrap resampling (1,000 replicates). Logistic regression provided a transparent baseline for comparison, facilitating the interpretation of feature importance and benchmarking performance gains achieved by additional machine learning algorithms. Feature selection and model explanation.

The SHAP (SHapley Additive exPlanations) (24) framework was integrated into the feature selection pipeline to objectively quantify variable importance and address model interpretability. Following LASSO-based preliminary screening, SHAP values were systematically calculated across all candidate models to rank features by their predictive contribution. A sequential backward elimination strategy was implemented: features were iteratively pruned in descending order of SHAP importance, while monitoring model performance via AUC stability. The elimination process terminated when a statistically significant decline in AUC (>5% relative reduction, *p* < 0.05 by Delong’s test) (25) indicated critical feature loss. This approach ensured retention of the optimal feature subset that maximized predictive capacity while minimizing redundancy.

### Statistical analysis

This study implemented the entire analytical workflow using R language (version 4.4.2). Methodological reliability was ensured through strict data isolation protocols. Prior to feature selection, stratified random sampling with 10 repeated splits was performed using the createDataPartition() function from the caret package, pre-partitioning the dataset into training (70%) and validation (30%) sets. This rigorous partitioning guaranteed the validation set remained completely isolated during the LASSO regression feature selection process, effectively eliminating data leakage risks. Continuous variables with skewed distributions were summarized using median and interquartile range (IQR). Between-group comparisons employed non-parametric tests: Mann–Whitney U test for two-group comparisons and Kruskal-Wallis H test for multi-group comparisons. Categorical variables were expressed as percentages (%) and analyzed using Pearson’s χ^2^ test or Fisher’s exact test, as appropriate for expected cell frequencies.

## Results

A total of 756 patients were included in this study, with 217 (28.7%) developing ENAD. The median age was 68.0 years (IQR: 57.0–76.0), and the male-to-female ratio was approximately 7:3. APACHE II scores were comparable between groups (median: 25.0 [IQR: 19.0–29.0], *p* = 0.406), indicating similar illness severity.

**Figure 2:**
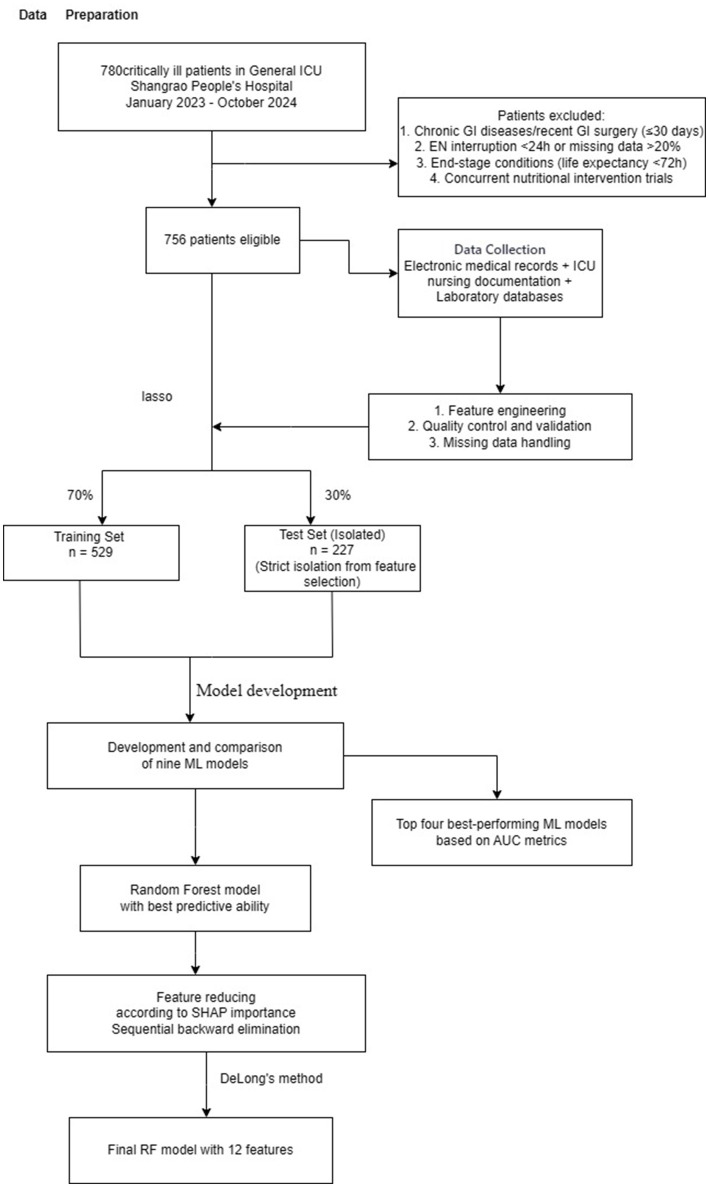
Study flowchart showing patient recruitment, data preparation, feature selection using LASSO regression, machine learning model development and validation for predicting enteral nutrition-associated diarrhea in ICU patients. ICU, intensive care unit; LASSO, least absolute shrinkage and selection operator; ML, machine learning; RF, random forest.

Significant differences were observed in treatment characteristics. Patients with ENAD more frequently received renal replacement therapy (34.6% vs. 25.6%, *p* = 0.017), prokinetics (55.3% vs. 29.7%, *p* < 0.001), probiotics (44.7% vs. 23.5%, *p* < 0.001), and sedatives (90.8% vs. 83.7%, *p* = 0.016). Nasoenteric feeding tubes were more common in the ENAD group (19.8% vs. 10.6%, *p* = 0.001), as was mixed feeding (47.5% vs. 25.4%, *p* < 0.001). Clinical course parameters differed significantly between groups. The ENAD group had longer mechanical ventilation duration (7.0 vs. 4.0 days, *p* < 0.001) and antibiotic therapy duration (16.0 vs. 10.0 days, *p* < 0.001). Serum sodium levels were slightly higher in the ENAD group (140.0 vs. 139.0 mmol/L, *p* = 0.048), while other laboratory parameters showed no significant differences. Notably, room temperature was lower in the ENAD group (20.0°C vs. 21.0°C, *p* < 0.001) (Table 1). Details of the study design are displayed in Figure 2.

### Model development and performance comparison

**Figure 3:**
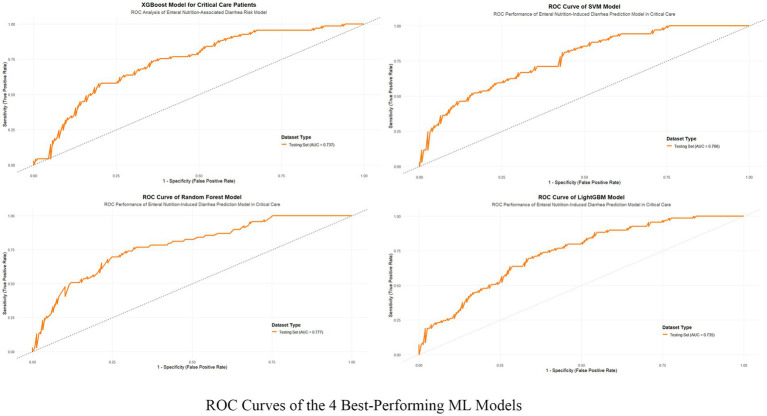
ROC curves of the 4 best-performing machine learning models. The figure illustrates the Receiver Operating Characteristic (ROC) curves of the four top-performing machine learning models—XGBoost, SVM, Random Forest, and LightGBM—that were applied to predict enteral nutrition-associated diarrhea in critical care patients. The true positive rate (sensitivity) is plotted against the false positive rate (1-specificity) for the test datasets of each model.

**Figure 4:**
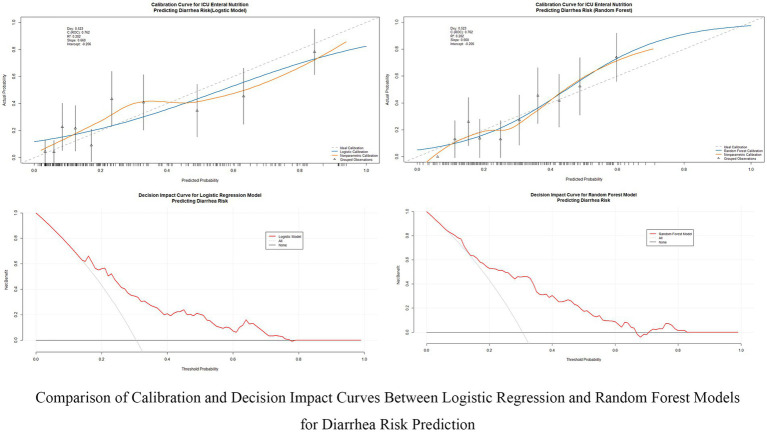
Comparison of calibration and decision impact curves between logistic regression and random forest models. This figure compares the calibration performance and clinical decision impact between the Logistic Regression and Random Forest models for predicting diarrhea risk in ICU patients receiving enteral nutrition. The calibration curves (top row) evaluate the alignment between predicted and actual probabilities, while the decision impact curves (bottom row) assess the clinical utility of these models across different decision thresholds.

This study evaluated the predictive performance of nine machine learning models for diarrhea risk prediction associated with enteral nutrition (EN). Table 2 presents the performance metrics for all nine models, while Figure 3 summarizes the performance of the top four models based on the area under the ROC curve (AUC). Feature contributions from the 18 variables are visualized in the SHAP summary plot (Supplementary Figure 2). Random forest outperformed other models in most metrics and emerged as the best predictive model. It achieved the highest AUC (0.777 [95% CI: 0.702–0.830]), recall (0.936 [95% CI: 0.897–0.987]), accuracy (0.787 [95% CI: 0.743–0.873]), and F1-score (0.835 [95% CI: 0.787–0.874]). The logistic regression model, used as the baseline approach, showed solid but comparatively lower performance with an AUC of 0.762 (95% CI: 0.695–0.828) and an F1-score of 0.817 (95% CI: 0.769–0.859). The second-best model was XGBoost, which had a slightly lower AUC (0.737 [95% CI: 0.668–0.803]) but demonstrated excellent recall (0.841 [95% CI: 0.781–0.898]) and comparable accuracy (0.730 [95% CI: 0.673–0.788]) to random forest. The support vector machine (SVM) and LightGBM were ranked third and fourth, respectively, with balanced AUCs (SVM: 0.766 [95% CI: 0.701–0.842]; LightGBM: 0.735 [95% CI: 0.667–0.735]) and high recall values (SVM: 0.780 [95% CI: 0.711–0.842]; LightGBM: 0.778 [95% CI: 0.714–0.842]). Figure 4 illustrates the calibration curves and decision-analytic curves (DACs) comparing the best model, random forest, with the baseline logistic regression model. Both models demonstrated good calibration, with predicted probabilities aligning closely with observed risks. However, the calibration curve for the random forest model (right panel) showed better agreement with the ideal diagonal line, particularly at higher predicted probabilities, suggesting stronger reliability. The DACs confirmed that the random forest model offers higher net benefits across all threshold probabilities when compared to logistic regression. This result highlights its superior utility in identifying clinical thresholds for decision-making and reducing unnecessary interventions.

**Table 2:** Performance metrics (95% CI) of machine learning models for predicting enteral nutrition-associated diarrhea risk in critically ill ICU patients.

Model	AUC (95% CI)	Recall (95% CI)	Accuracy (95% CI)	F1Score (95% CI)	Precision (95% CI)	NPV (95% CI)
Logistic	0.762 (0.695–0.828)	0.784 (0.718–0.841)	0.735 (0.677–0.787)	0.817 (0.769–0.859)	0.853 (0.796–0.904)	0.784 (0.718–0.842)
SVM	0.766 (0.701–0.766)	0.780 (0.717–0.842)	0.748 (0.690–0.805)	0.830 (0.786–0.874)	0.885 (0.834–0.934)	0.754 (0.687–0.813)
Random Forest	0.777 (0.702–0.830)	0.936 (0.897–0.968)	0.743 (0.673–0.787)	0.835 (0.787–0.873)	0.754 (0.687–0.807)	0.677 (0.515–0.833)
XGBoost	0.737 (0.668–0.803)	0.841 (0.781–0.898)	0.730 (0.673–0.788)	0.812 (0.763–0.855)	0.786 (0.720–0.845)	0.569 (0.444–0.692)
LightGBM	0.735 (0.667–0.735)	0.778 (0.714–0.842)	0.695 (0.637–0.752)	0.781 (0.730–0.830)	0.783 (0.719–0.846)	0.778 (0.713–0.844)
Neural Network	0.757 (0.709–0.800)	0.793 (0.725–0.865)	0.715 (0.692–0.742)	0.800 (0.773–0.826)	0.814 (0.770–0.860)	0.814 (0.770–0.860)
AdaBoost	0.730 (0.656–0.800)	0.830 (0.765–0.886)	0.725 (0.659–0.779)	0.806 (0.756–0.861)	0.787 (0.718–0.846)	0.558 (0.433–0.683)
DT	0.732 (0.660–0.797)	0.905 (0.859–0.946)	0.726 (0.668–0.782)	0.821 (0.776–0.864)	0.752 (0.692–0.814)	0.596 (0.438–0.755)
Naive Bayes	0.742 (0.664–0.812)	0.246 (0.158–0.350)	0.752 (0.690–0.805)	0.378 (0.257–0.350)	0.810 (0.637–0.955)	0.746 (0.682–0.802)

### Identification of the final model

The random forest model was identified as the optimal predictive model for this study. To evaluate feature importance and refine the model, SHAP values were applied iteratively to reduce three features at a time. The full 18-feature model achieved an AUC of 0.777 (95% CI: 0.702–0.830), whereas a simplified 3-feature model showed a significantly reduced AUC of 0.699 (95% CI: 0.626–0.772), with a statistically significant difference compared to the 18-feature model (*∆AUC = 0.078, p = 0.031*).

No significant differences in AUC were observed between models using 18, 15, and 12 features (*∆AUC = 0.012, p = 0.623* and *∆AUC = 0.023, p = 0.332*, respectively). However, significant performance differences were noted when comparing models with 6 features, 9 features, and 12 features, with all *p* values < 0.05.

Based on these findings, the random forest model using 12 features emerged as the optimal balance between predictive performance and feature parsimony. The final 12-feature random forest model achieved an AUC of 0.754 (95% CI: 0.685–0.823). These results suggest that the 12-feature model retains strong predictive capability while reducing the overall feature count, enhancing interpretability and potential applicability in clinical settings.

### Model explanation

**Figure 5:**
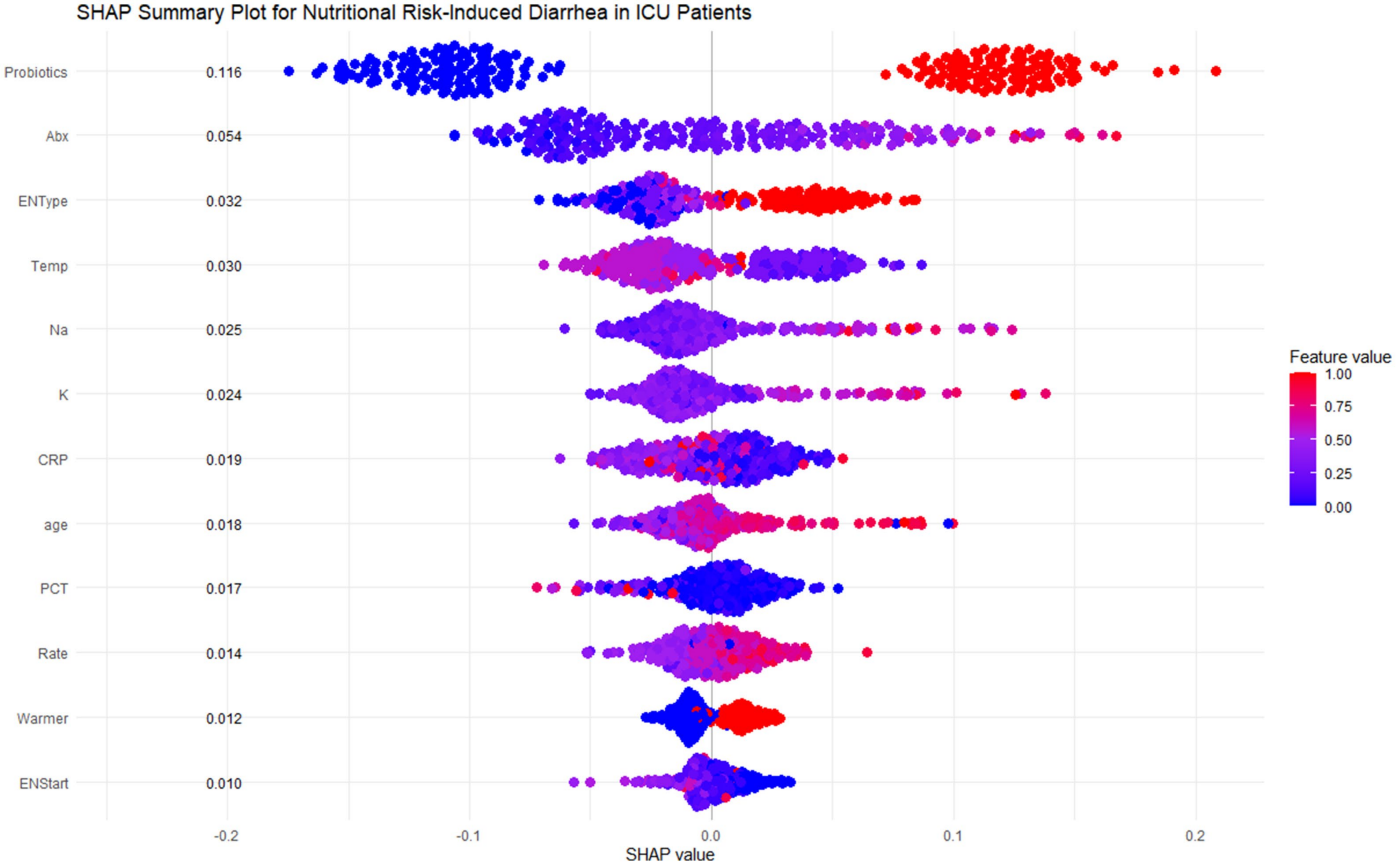
SHAP summary plot for nutritional risk-induced diarrhea in ICU patients. This figure shows the SHAP summary plot for the Random Forest model, highlighting the feature importance and individual contributions of the 12 most influential features in predicting diarrhea risk for ICU patients. Each row corresponds to a specific feature, and each dot represents a single patient. The x-axis shows the SHAP values, which measure the magnitude and direction of the feature’s effect on the prediction. The color of each dot indicates the actual feature value for the corresponding patient, with red representing higher values and blue representing lower values.

SHAP analysis was employed to interpret the random forest model, highlighting feature contributions to ENAD predictions. The SHAP summary plot (Figure 5) shows ranked contributions based on average SHAP values. Probiotics had the highest contribution (mean SHAP value: 0.116), followed by antibiotic use duration and enteral nutrition type. Specialized or mixed EN formulas contributed more to risk than standard formulas.

Key predictive patterns emerged across multiple domains. Environmental factors showed that lower ambient temperatures (<20°C) increased diarrhea risk. Laboratory parameters demonstrated that high sodium levels (>145 mmol/L) significantly increased risk, while higher potassium levels (>5.5 mmol/L) were protective. Age showed positive correlation, with patients >60 years contributing more to predictions. CRP exhibited complex patterns where lower levels (<100 mg/L) increased risk while higher values appeared protective. Additional factors including PCT, infusion rate, warming devices, and early EN initiation (<6 days) showed smaller but relevant contributions.

**Figure 6:**
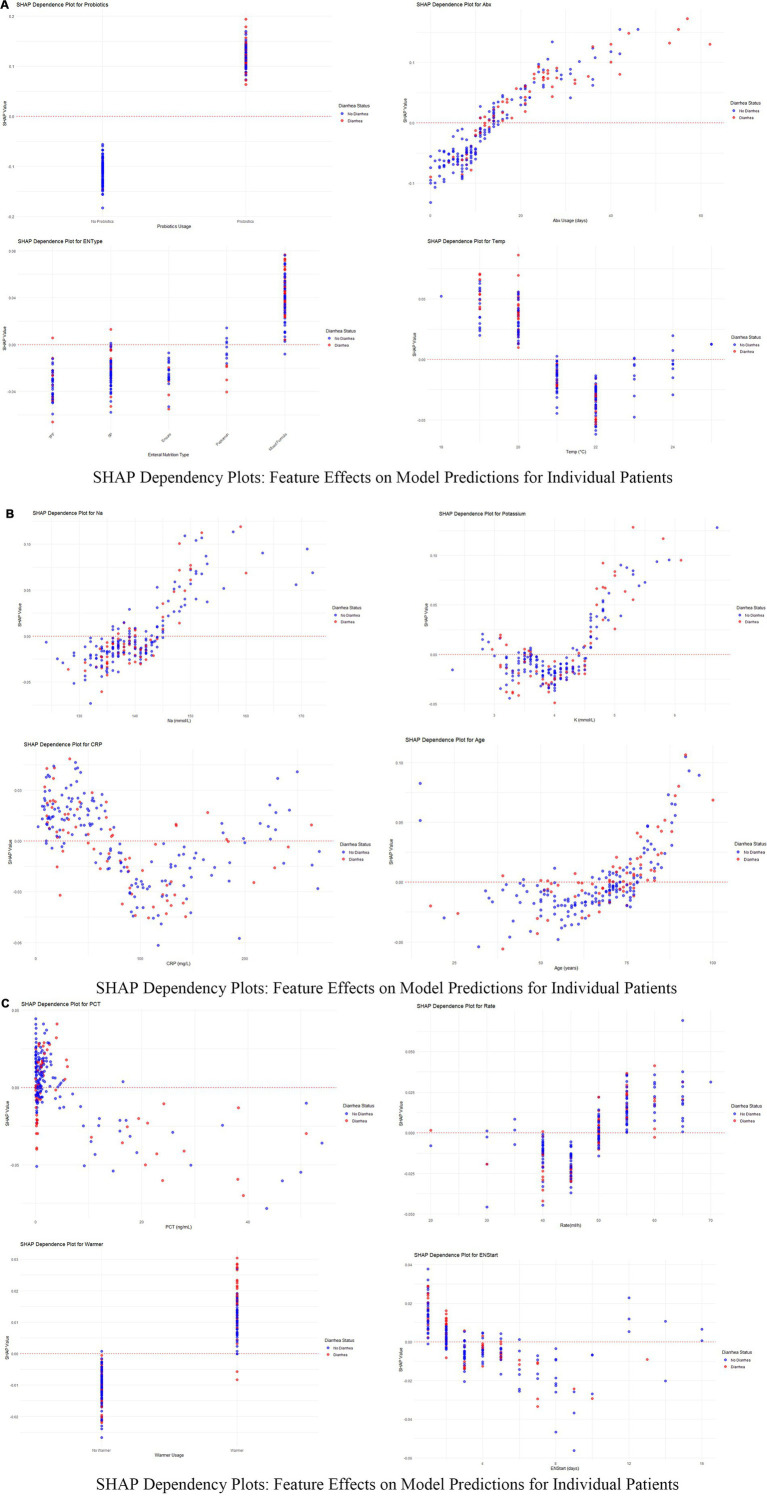
**(A–C)** SHAP dependency plots for key features in predicting diarrhea risk. These figures present the SHAP dependency plots for several critical features in the Random Forest model, demonstrating how changes in feature values affect model predictions. Each plot reflects the relationship between a feature’s value (x-axis) and its SHAP value (y-axis) for individual patients. Points are color-coded to represent feature values across patients, with red indicating higher values and blue indicating lower values. Points are vertically stacked to show density and to reveal patient-specific SHAP value distributions.

**Figure 7:**
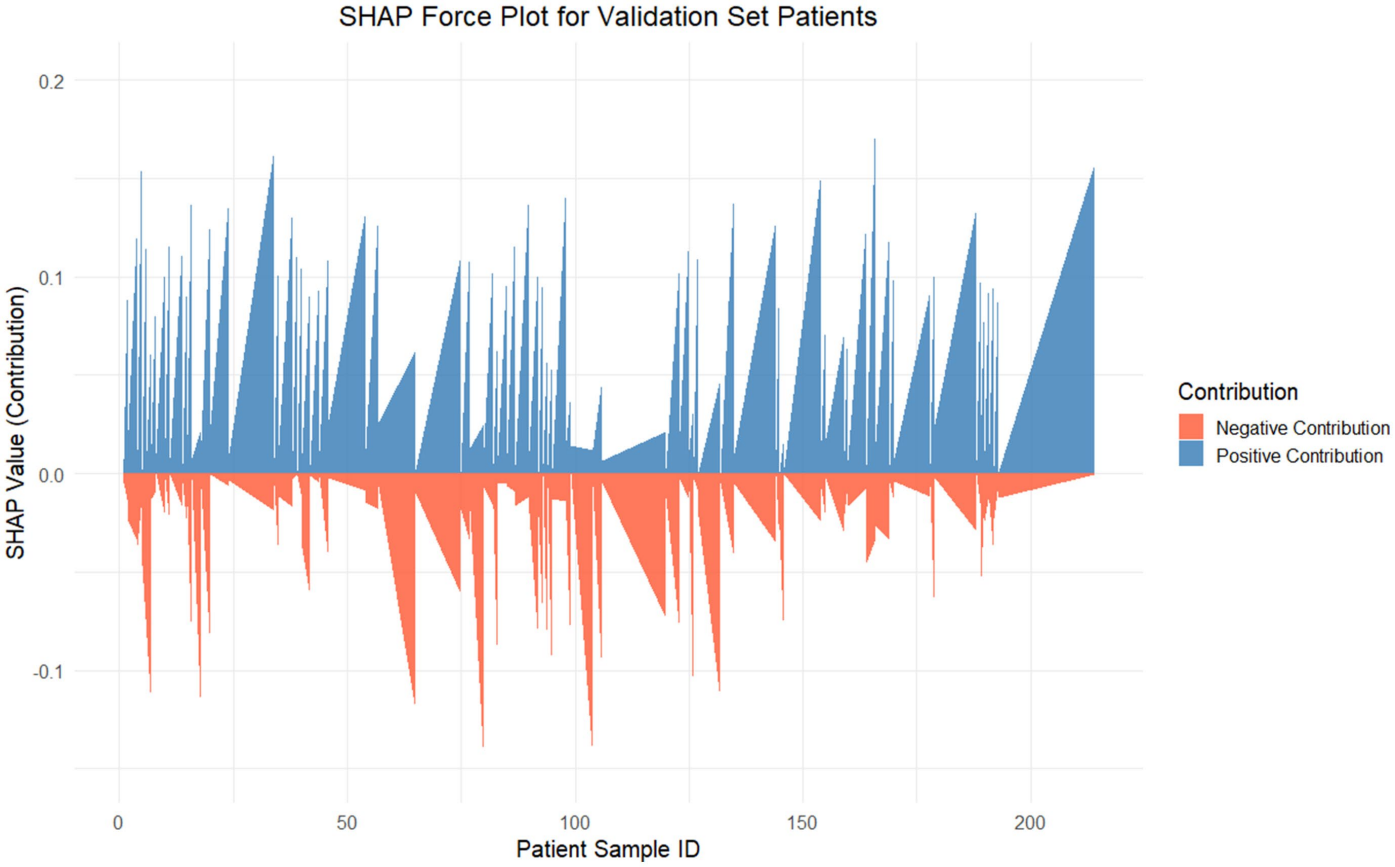
SHAP force plot for internal validation. This figure visualizes the SHAP values for each patient in the internal validation set, showcasing the contributions of features to the Random Forest model’s predictions. The SHAP value (y-axis) represents the contribution magnitude of all features to the predictive score for each patient (x-axis). The positive contributions (blue) push the prediction toward a higher risk, whereas the negative contributions (red) reduce the predicted risk.

Dependency plots (Figures 6A–C) illustrated the relationships between feature values and predictive outputs, providing insights into how individual features influenced model predictions. Individual patient analysis using SHAP force plots (Figure 7) further visualized feature contributions for each patient, where positive values (blue bars) enhanced predicted risk and negative values (red bars) reduced risk. This approach provided intuitive understanding of the model’s decision-making process at the individual level.

## Discussion

This study explored the predictive factors and model development for assessing diarrhea risk in ICU patients receiving enteral nutrition (EN), ultimately identifying a random forest model as the optimal choice with superior predictive performance and clinical applicability. A total of 756 ICU patients were analyzed, among whom 217 (28.7%) experienced diarrhea as defined by standard criteria. The analysis revealed significant differences in clinical and therapeutic variables between the diarrhea and non-diarrhea groups. Probiotics, duration of antibiotic therapy, and the use of mixed feeding formulas emerged as the strongest predictors of diarrhea, as identified by SHAP analysis, highlighting their critical role in influencing patient outcomes. Probiotics had the highest mean SHAP contribution (0.116), indicating that they significantly increased predicted diarrhea risk, especially in cases of high usage (26, 27). Similarly, prolonged antibiotic therapy (>16 days in the diarrhea group vs. 10 days in the non-diarrhea group, *p* < 0.001) showed a clear positive association with increased risk, emphasizing the need for cautious antibiotic stewardship in ICU settings. Mixed feeding formulas were also more frequent in the diarrhea group (47.5% vs. 25.4%, *p* < 0.001), further contributing to diarrhea prediction in patients receiving EN (28). Beyond treatment-related factors, specific environmental and laboratory parameters showed significant associations with diarrhea risk. Lower room temperatures (<20°C) were found to increase diarrhea predictions significantly, underlining the role of environmental interventions in managing risk for ICU patients (29). Serum sodium levels in the diarrhea group were slightly higher (140 mmol/L vs. 139 mmol/L, *p* = 0.048) and were associated with positive SHAP values, indicating its contribution to higher risk when sodium exceeded 145 mmol/L. Potassium exhibited a non-linear pattern, with moderate levels (4–5 mmol/L) contributing positively to diarrhea risk, while higher levels (>5.5 mmol/L) had a protective effect (30). Interestingly, the age of the patients was another significant factor, with older age (≥60 years) correlating with higher risk, suggesting that this patient subgroup requires closer monitoring.

Clinical interpretation of key predictive factors reveals important insights for ENAD prevention. Analysis of dependency relationships identified critical determinants requiring targeted nursing interventions. Probiotics showed the strongest association with diarrhea risk, challenging conventional assumptions about their protective effects in critically ill patients. This counterintuitive finding likely reflects selection bias, where probiotics are preferentially administered to patients already at high gastrointestinal risk, or indicates inappropriate strain selection for ICU populations (31, 32). Prolonged antibiotic use demonstrated a clear dose–response relationship, with durations exceeding 30 days substantially increasing risk, emphasizing the critical importance of antibiotic stewardship to minimize gut dysbiosis (33). Mixed feeding formulations carried significantly higher risk than standard approaches, reflecting their complex gastrointestinal impact in vulnerable patients.

Environmental and physiological factors provided actionable clinical insights. Ambient temperature emerged as a modifiable risk factor, with temperatures below 20°C significantly increasing diarrhea risk, while maintaining temperatures above 22°C proved protective. Electrolyte imbalances, particularly elevated sodium levels (>145 mmol/L), strongly predicted diarrhea development through hypernatremia-induced gut permeability and osmotic disruption (34). Potassium displayed complex patterns where moderate levels (4–5 mmol/L) increased risk, while higher concentrations (>5.5 mmol/L) were protective (35), suggesting optimal electrolyte balance is crucial for gastrointestinal stability.

Inflammatory markers revealed nuanced relationships requiring careful clinical interpretation. Moderate CRP levels (~100 mg/L) posed greatest risk, while both low (<50 mg/L) and very high levels (>150 mg/L) were associated with reduced predictions (36), suggesting that mild-to-moderate inflammation disrupts gut integrity more than severe inflammatory states. Similarly, elevated PCT levels (>10 ng/mL) increased risk due to systemic inflammation or sepsis, while lower levels had minimal impact (37). Age-related vulnerability was evident, with patients over 60 years showing markedly increased risk due to reduced gut motility, immune dysfunction, and comorbidity burden (38, 39).

Feeding-related factors highlighted opportunities for targeted interventions. Rapid feeding rates exceeding 50 mL/h correlated with increased risk due to gastrointestinal intolerance, while moderate rates (30–40 mL/h) had negligible effects (40). Delayed EN initiation beyond 8 days significantly increased risk through gut disuse effects, while early initiation (≤4 days) proved protective by maintaining gut integrity and reducing bacterial overgrowth. Warmer usage introduced slightly elevated risks, potentially through indirect metabolic or fluid balance effects, though this association requires further investigation. These findings emphasize evidence-based preventive strategies: maintaining optimal room temperatures (21–23°C), implementing enhanced electrolyte monitoring for sodium levels >142 mmol/L, questioning probiotic use in high-risk patients, advocating for antibiotic de-escalation when appropriate, using standard formulations when feasible, maintaining feeding rates below 50 mL/h for vulnerable patients, and prioritizing early EN initiation. These data-driven approaches highlight the multifactorial nature of diarrhea risk and support individualized patient management in ICU settings.

This prediction model of random forest developed in the present study could be implemented into clinical practise based on step-by-step strategy to reduce and prevent enteral nutrition-associated diarrhea with higher clinical efficacy. A 12-feature model (AUC 0.754; 95% CI 0.685–0.823) preserved predictive prowess while reducing to make the model feasible in clinical practice and incorporated into the intensive care information systems as an early warning score for temperature. Steps for the Implementation pathway: First hospitals may adopt Electronic Health Records derived decision support tools to extract the major predictive variables (e.g., duration of antibiotic use, probiotic use, enteral nutrition type ambient temperature and electrolyte levels) automatically; Second, the prediction results are divided (high, medium and low risk) for each patient with customized risk-stratified intervention recommendations (Figure 3) looking at SHAP value analysis; Third, real-time risk assessment to generate alerts could be built into mobile phone applications to allow healthcare providers to modulate their treatment plans in real-time; Last, preventive response protocols ought to be established in place to initiate chances measures for those who are at very high risk with high-risk strategies like 2-times probiotic dosage, regulation of room temperature variation, monitoring electrolytes and rationalisation of antibiotics. Prospective validation of the model during implementation should refine algorithm performance, and training healthcare staff to understand and appropriately use model output should be ensured. This should be noted, however this model is not meant to replace clinical experience but rather something that we use as a companion in making the final decision on what is best for our patient (41). By incorporating this predictive model into the daily medical routine, clinical teams would allow to detect shortly at risk patients and intervene at time by providing specific preventive measures, slims down diarrhea prevalence significantly to reduce mechanical ventilation time, enhance enteral nutrition support and lastly prognosis with high-quality living among critically ill patients (42).

The developed RF prediction model provides significant opportunities for integration into daily ICU nursing practice through comprehensive workflow enhancement and clinical decision support systems. Early Risk Identification and Proactive Interventions: The 12-variable model enables ICU nurses to identify patients at high risk for ENAD within the first 24–48 h of ICU admission, facilitating timely implementation of evidence-based preventive interventions. ICU nurses can utilize the model’s output to stratify patients into different ENAD risk categories (high, medium, low), which facilitates more proactive care approaches and prioritizes patients requiring intensive monitoring. For high-risk patients identified by the model, nurses can implement enhanced assessment protocols including hourly stool monitoring, detailed documentation of feeding tolerance, and early consultation with nutrition specialists when appropriate.

### Enhanced nursing decision support for targeted interventions

The model’s SHAP-based feature importance provides nurses with actionable clinical insights to guide specific interventions. Based on the model’s predictions, nurses can make informed decisions regarding feeding regimen adjustments, such as reducing feeding rates below 50 mL/h for vulnerable patients or advocating for standard formulations over mixed feeding approaches when clinically appropriate. The model’s identification of critical electrolyte thresholds enables nurses to implement targeted monitoring protocols, with closer electrolyte monitoring particularly focused on sodium levels exceeding 142 mmol/L and potassium levels in the 4–5 mmol/L range. Environmental interventions, particularly maintaining optimal room temperatures (21–23°C), can be directly implemented by nursing staff based on the model’s environmental risk factors, representing a simple yet effective nursing intervention.

### Electronic health record integration and clinical decision support

The model’s risk scoring can be integrated with electronic health record (EHR) systems to calculate risk scores during data entry, providing automated alerts on nursing dashboards and bedside monitors. This integration reduces cognitive burden while enhancing patient safety through real-time risk assessment capabilities that enable nurses to receive instant notifications when patient parameters change, allowing for immediate intervention adjustments. The system can generate evidence-based care protocols and nursing care plans tailored to individual risk profiles, supporting standardized yet personalized care approaches. Mobile application integration allows bedside nurses to access risk assessments and intervention recommendations in real-time, facilitating immediate clinical decision-making during patient care activities.

### Professional role enhancement in interdisciplinary care

This predictive tool significantly empowers ICU nurses during interprofessional rounds by providing objective, data-driven insights that strengthen their clinical voice and decision-making authority. The model assists in clinical decision-making by providing nurses with additional risk assessment information during interprofessional rounds, which can inform discussions about probiotic use, antibiotic duration, and early consultation with dietitians when appropriate. Nurses can present concrete risk assessments and evidence-based intervention recommendations, reinforcing their critical role in risk prevention and patient advocacy. The model supports nursing documentation through structured risk assessment frameworks, improving the quality and completeness of nursing records while demonstrating the impact of nursing interventions on patient outcomes.

Implementation of this predictive tool contributes to nursing practice by providing additional data to support interventions, improving documentation through structured risk assessment, and helping identify patients who may benefit from closer monitoring (43). The model can be included in nursing education programs to help ICU nurses understand the various factors contributing to ENAD risk and develop competency in using predictive analytics for clinical decision-making. Additionally, the tool supports quality improvement efforts by enabling nurses to monitor prevention strategies, identify care patterns, and evaluate intervention effectiveness across patient populations. The comprehensive integration approach transforms the predictive model from a passive tool into an active component of nursing practice, enhancing both the quality of patient care and the professional development of ICU nursing staff. However, it should be emphasized that this model serves as a clinical decision support tool to augment, not replace, nursing clinical judgment and expertise, with further validation and real-world testing needed to fully understand its practical utility and limitations in diverse ICU settings.

### Limitations

Our study attempts to contribute to the understanding of risk factors potentially associated with diarrhea in ICU patients receiving enteral nutrition, though several limitations should be carefully considered when interpreting these findings.

First, the retrospective design of the analysis may introduce selection bias, limiting our ability to draw conclusions about causal relationships concerning diarrheal risk and other identified factors. Additionally, the retrospective nature introduces potential documentation bias, as the completeness and accuracy of medical records may vary across different healthcare providers and time periods. Missing or inconsistently recorded data could systematically impact our model’s performance and generalizability.

Second, several key clinical variables that may significantly influence ENAD development were underrepresented or unavailable in our dataset. These include detailed information on enteral nutrition delivery methods (continuous vs. bolus feeding), specific feeding tube positioning and functionality, concurrent medication effects (particularly prokinetic agents, antibiotics, and proton pump inhibitors), detailed fluid balance records, and patient-specific factors such as pre-existing gastrointestinal conditions and fluctuations in illness severity during ICU stay. The absence of these variables may limit the model’s predictive accuracy and clinical applicability.

Third, even though the predictability provided by SHAP analysis is intuitive, it will likely not account for all the complex relationships and interactions among variables that may lead to misunderstandings about the genesis of these behaviors. Moreover, SHAP explanations, despite their mathematical rigor, require careful clinical contextualization to be translated into actionable nursing interventions. The current analysis may oversimplify the multifactorial nature of ENAD development and could potentially mislead clinical decision-making if applied without appropriate clinical judgment and validation.

Fourth, the study is single-center, which limits the generalizability of our findings to other ICU cohorts or patient types. Multi-center studies are needed to validate the model in different contexts. Our findings may not apply to ICUs with different patient populations, feeding protocols, staffing patterns, or technological infrastructures. The institution-specific practices and patient demographics at our center may constrain the external validity of the predictive model.

Fifth, the practical implementation of real-time model deployment presents significant challenges that were not fully addressed in this study. These include integration with existing electronic health record systems, computational requirements for continuous prediction updates, staff training needs for model interpretation, and the development of standardized protocols for responding to model predictions. The feasibility and cost-effectiveness of implementing such a system in routine clinical practice require further investigation.

Sixth, the model was developed and validated exclusively within a Chinese ICU setting, which may significantly limit its applicability to international healthcare environments with different patient populations, clinical practices, and healthcare delivery systems. Cultural dietary patterns, genetic variations in drug metabolism, and population-specific comorbidity profiles could substantially influence ENAD development patterns and may not be adequately represented in our model. Furthermore, variations in ICU management protocols, nursing-to-patient ratios, enteral nutrition formulation standards, and antibiotic prescribing practices across different countries and healthcare systems could impact the model’s predictive performance when applied outside the Chinese context. International validation studies are essential to assess the model’s transferability across diverse populations and healthcare settings. Multi-center international trials involving ICUs from different continents would be particularly valuable to evaluate whether the identified risk factors maintain their predictive value across varied clinical contexts, patient ethnicities, and institutional practices. Such studies should specifically examine whether the relative importance of predictive variables (particularly environmental factors like room temperature, feeding protocols, and probiotic usage patterns) remains consistent across different healthcare infrastructures and clinical cultures. Additionally, the development of region-specific model adaptations or recalibration strategies may be necessary to ensure optimal performance in non-Chinese ICU environments, requiring collaborative international research networks and standardized data collection protocols to facilitate meaningful cross-cultural validation and implementation.

Finally, there are factors of interest that were not included in our analysis, which may impact the outcomes (e.g., form and composition of EN formulations, fluid management strategies, and/or other co-morbidities). The temporal dynamics of patient conditions and treatment responses, which may significantly influence ENAD development, were not adequately captured in our static modeling approach. Even if our results are robust, prospective studies and clinical trials are required to support these results, as well as to implement them through more concrete interventions and guidelines that can be applied in daily clinical practice for improved patient outcomes. Future research should focus on external validation across multiple centers, prospective model testing, and the development of implementation frameworks that address the technological, educational, and workflow integration challenges identified in this study.

## Conclusion

This study explored factors influencing diarrhea risk in ICU patients receiving enteral nutrition (EN) and developed a predictive model based on machine learning techniques. A total of 756 patients, including 217 who developed diarrhea, were analyzed, revealing key clinical and therapeutic factors such as probiotics, prolonged antibiotic use, mixed feeding formulas, and environmental and laboratory parameters (e.g., serum sodium, potassium, CRP, and room temperature) contributing to diarrhea risk. Nine machine learning algorithms were compared, using logistic regression as the baseline model. Random forest was identified as the most suitable model due to its balance between predictive performance and clinical applicability, achieving an AUC of 0.777 with acceptable recall, accuracy, and F1-score. The model was further refined using SHAP-based feature selection, retaining 12 essential features to optimize predictive power while enhancing interpretability. The SHAP analysis clarified the individual contributions and nonlinear relationships of predictive features, offering insights into risk dynamics under different clinical conditions. While the random forest model showed good performance in identifying diarrhea risk, further validation and prospective studies are needed to confirm these findings and enhance their applicability in varied clinical settings.

## Data Availability

The raw data supporting the conclusions of this article will be made available by the authors, without undue reservation.

## Supplementary material

SUPPLEMENTARY FIGURE 1 This figure displays the occurrence of diarrhea among patients with different comorbidity types. Chi-square test results (χ² = 11.01, *p* = 0.357) indicate no statistically significant correlation between comorbidity type and diarrhea occurrence. The data shows that patients with tumor-related diseases had the highest proportion of diarrhea (38.1%), followed by those with cardiovascular and cerebrovascular diseases (34.6%) and patients without complications (33.3%), while patients with respiratory and digestive system diseases had the lowest incidence of diarrhea (21.4%).

SUPPLEMENTARY FIGURE 2 SHAP summary plot for 18 key features identified by LASSO. This figure summarizes the SHAP analysis for the 18 features identified by LASSO regression as critical predictors of nutritional risk-induced diarrhea in ICU patients. Each dot represents a single patient, and the x-axis displays the SHAP value, indicating the magnitude and direction of each feature's contribution to the model's prediction. Features are ranked by their importance, with those having the highest mean absolute SHAP values appearing at the top. The color gradient represents the actual feature values for patients, where red denotes higher feature values and blue denotes lower feature values.
