# Supplementary figures and images for: Machine learning-based predictive model for enteral nutrition-associated diarrhea in ICU patients and its nursing applications
Source: Front Nutr. 2025 Jun 25;12:1584717. doi: 10.3389/fnut.2025.1584717 (PMC12237648; doi:10.3389/fnut.2025.1584717)

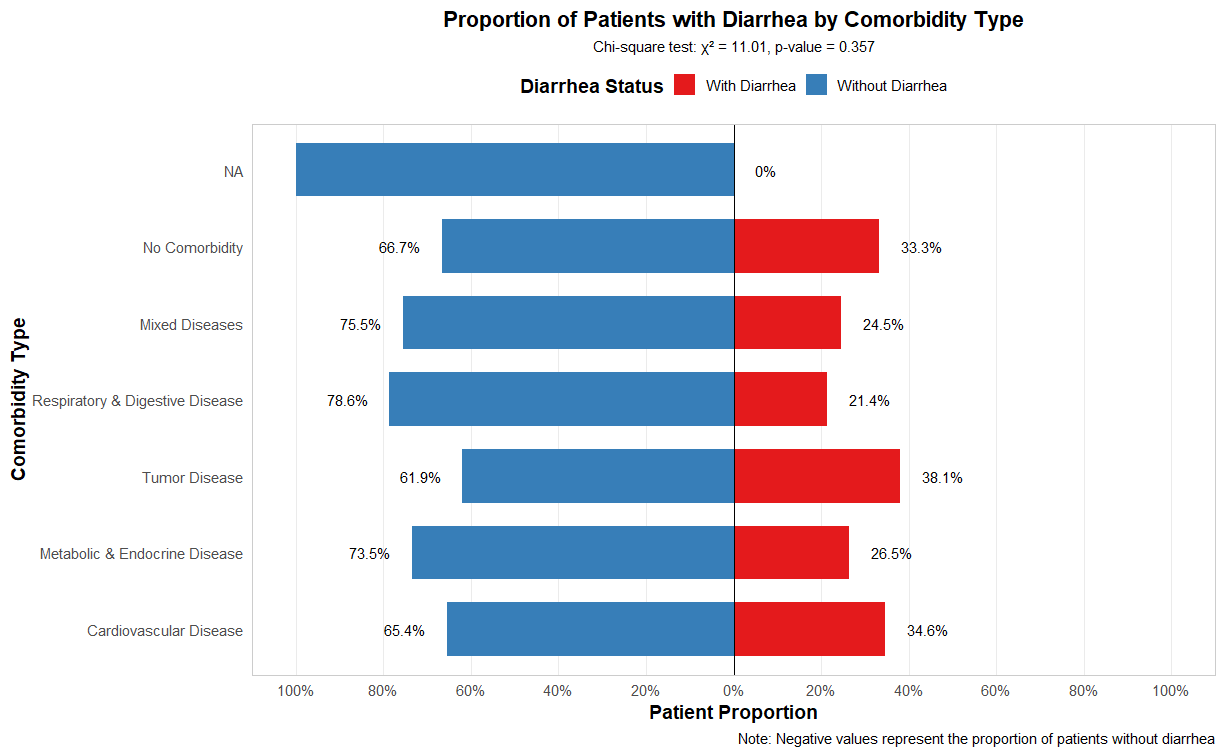

Supplement: SUPPLEMENTARY FIGURE 1 — This figure displays the occurrence of diarrhea among patients with different comorbidity types. Chi-square test results (χ² = 11.01, p = 0.357) indicate no statistically significant correlation between comorbidity type and diarrhea occurrence. The data shows that patients with tumor-related diseases had the highest proportion of diarrhea (38.1%), followed by those with cardiovascular and cerebrovascular diseases (34.6%) and patients without complications (33.3%), while patients with respiratory and digestive system diseases had the lowest incidence of diarrhea (21.4%). [file Image_1.tiff]

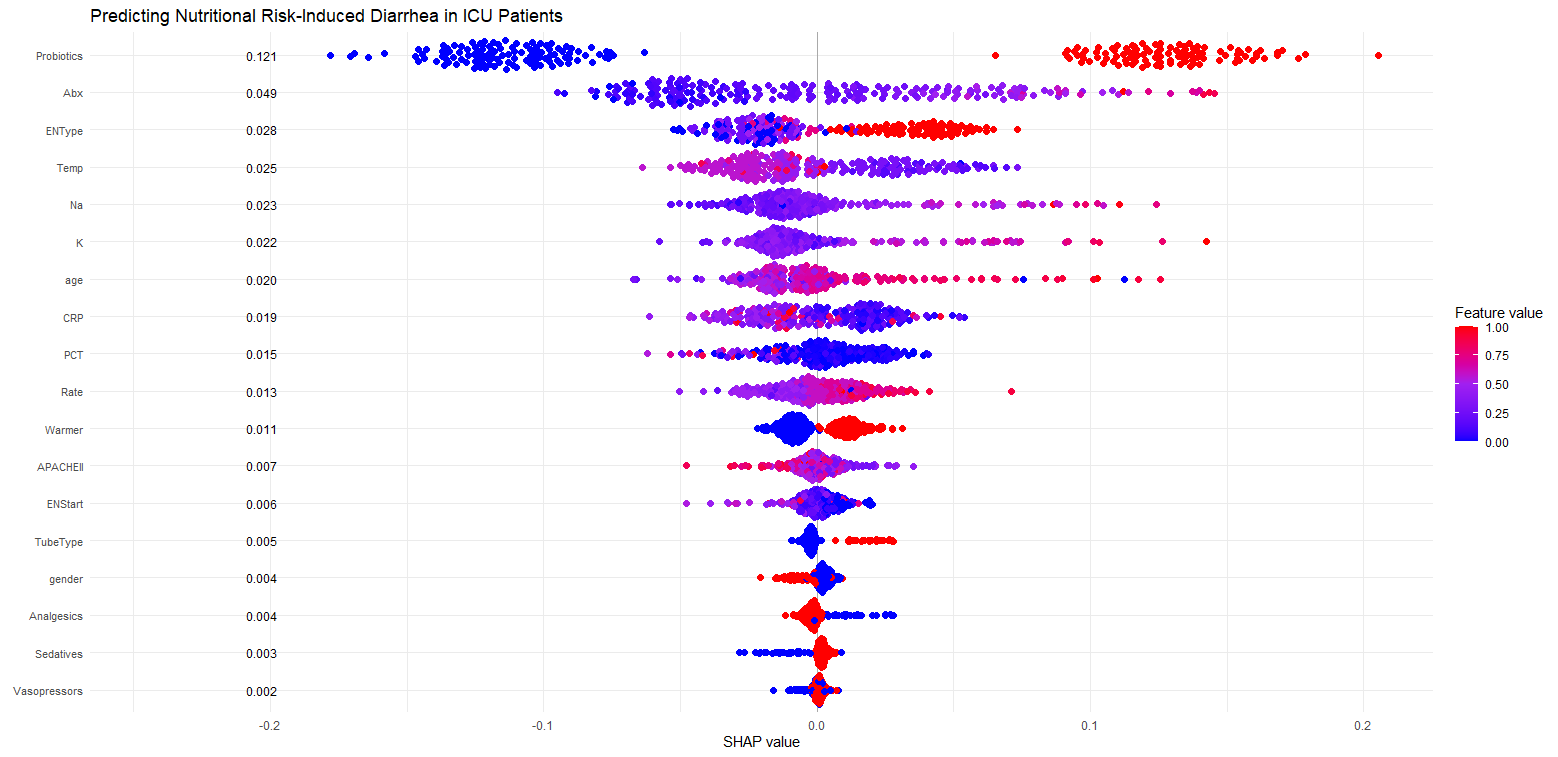

Supplement: SUPPLEMENTARY FIGURE 2 — SHAP summary plot for 18 key features identified by LASSO. This figure summarizes the SHAP analysis for the 18 features identified by LASSO regression as critical predictors of nutritional risk-induced diarrhea in ICU patients. Each dot represents a single patient, and the x-axis displays the SHAP value, indicating the magnitude and direction of each feature's contribution to the model's prediction. Features are ranked by their importance, with those having the highest mean absolute SHAP values appearing at the top. The color gradient represents the actual feature values for patients, where red denotes higher feature values and blue denotes lower feature values. [file Image_2.tiff]
