# Supplementary material for: Machine learning-based predictive model for enteral nutrition-associated diarrhea in ICU patients and its nursing applications
Source: Front Nutr. 2025 Jun 25;12:1584717. doi: 10.3389/fnut.2025.1584717 (PMC12237648; doi:10.3389/fnut.2025.1584717)
Supplement: Supplementary file 3 [file Table_1.docx]

**Hyperparameter Settings and Grid Search Ranges for Machine Learning Models**

**SVM Hyperparameter Search Range and Optimization Results**
**Hyperparameter Search Range**
*cost* (regularization parameter)
Search range: 0.01 (fixed)

*gamma* (kernel parameter)
Search range: 0.001 (fixed)

*kernel* (kernel type)
Search range: {"linear"}

**Optimal Values**

cost = 0.01

gamma = 0.001

kernel = "linear"

**Random Forest Hyperparameter Grid Search Range and Optimal Values：**

*mtry* (number of variables selected for each split)
Search range: {1, 2, 3, 4, 5}

*min.node.size* (minimum sample size for leaf nodes)
Search range: {1, 5, 10}

*splitrule* (split rule)
Search range: {"gini", "extratrees"}

**Optimal Values**

mtry = 5

min.node.size = 1

splitrule = "gini"

**Validation Performance**

Classification accuracy: 74.51% (best performance across tuning parameters)

Kappa: 0.244 (best performance)

Validation protocol: 10-fold cross-validation

**XGBoost Hyperparameter Search Range and Optimization Results
Hyperparameter Search Range:**

*nrounds* (number of boosting rounds)
Search range: 100 (fixed)

*max_depth* (maximum tree depth)
Search range: 6 (fixed)

*eta* (learning rate)
Search range: 0.3 (fixed)

*gamma* (minimum loss reduction)
Search range: 0.2 (fixed)

*colsample_bytree* (subsample ratio of columns)
Search range: 1 (fixed)

*min_child_weight* (minimum sum of instance weights)
Search range: 2 (fixed)

*subsample* (subsample ratio of training instances)
Search range: 0.6 (fixed)

**Optimal Values**

nrounds = 100

max_depth = 6

eta = 0.3

gamma = 0.2

colsample_bytree = 1

min_child_weight = 2

subsample = 0.6

**LightGBM Hyperparameter Search Range and Optimization Results**
**Hyperparameter Search Range**
*obnum_leaves (number of leaves in one tree)
Search range: 20 (fixed)*

*learning_rate (shrinkage rate)
Search range: 0.3 (fixed)*

*max_depth (maximum tree depth)
Search range: 7 (fixed)*

*min_child_samples (minimum data in one leaf)
Search range: 50 (fixed)*

*feature_fraction (random feature selection ratio)
Search range: 0.8 (fixed)*

*bagging_fraction (data sampling ratio)
Search range: 0.6 (fixed)*

***Optimal Values***

*num_leaves = 20*

*learning_rate = 0.3*

*max_depth = 7*

*min_child_samples = 50*

*feature_fraction = 0.8*

*bagging_fraction = 0.6*

**Neural Network Hyperparameter Search Range and Optimization Results**
**Hyperparameter Search Range**
*size* (number of hidden layer nodes)
Search range: {1, 3, 5, 10}

*decay* (weight decay)
Search range: {0, 0.001, 0.01}

**Optimal Values**

size = 1

decay = 0.01

**AdaBoost Hyperparameter Search Range and Optimization Results**
**Hyperparameter Search Range**
*iter* (number of boosting iterations)
Search range: 50 (fixed)

*maxdepth* (maximum tree depth)
Search range: 2 (fixed)

*nu* (shrinkage parameter)
Search range: 1 (fixed)

**Optimal Values**

在·iter = 50

maxdepth = 2

nu = 1

**Decision Tree Hyperparameter Search Range and Optimization Results**
**Hyperparameter Search Range**
*cp* (complexity parameter)
Search range: {0.01, 0.02, 0.03, 0.04, 0.05, 0.06, 0.07, 0.08, 0.09, 0.10}

*minsplit* (minimum observations for a split)
Search range: 10 (fixed)

*maxdepth* (maximum tree depth)
Search range: 5 (fixed)

**Optimal Values**

cp = 0.02

minsplit = 10

maxdepth = 5

**Extra Trees Hyperparameter Search Range and Optimization Results**
**Hyperparameter Search Range**
*mtry* (number of features to consider at each split)
Search range: 4 (fixed)

*splitrule* (splitting rule)
Search range: {"extratrees"}

*min.node.size* (minimum node size)
Search range: 5 (fixed)

**Optimal Values**

mtry = 4

splitrule = "extratrees"

min.node.size = 5

**Naive Bayes Hyperparameter Search Range and Optimization Results**
**Hyperparameter Search Range**
*fL* (Laplace smoothing parameter)
Search range: 0 (fixed)

*usekernel* (kernel density estimation)
Search range: TRUE (fixed)

*adjust* (bandwidth adjustment for kernel density)
Search range: 2 (fixed)

**Optimal Values**

fL = 0

usekernel = TRUE

adjust = 2
