# Supplementary material for: Machine learning-based predictive model for enteral nutrition-associated diarrhea in ICU patients and its nursing applications
Source: Front Nutr. 2025 Jun 25;12:1584717. doi: 10.3389/fnut.2025.1584717 (PMC12237648; doi:10.3389/fnut.2025.1584717)
Supplement: Supplementary file 4 [file Table_2.docx]

| Variable Selection and Coefficient Estimation for Enteral Nutrition-Associated Diarrhea Model | | | | |
| --- | --- | --- | --- | --- |
| **No.** | **Variable** | **LASSO Coefficient** | **Selection Status** | **Clinical Effect** |
| 1 | (Intercept) | -3.541184 |  |  |
| 2 | gender | -0.262011 | Selected | Protective Factor |
| 3 | age | 0.003373 | Selected | Risk Factor |
| 4 | APACHEII | -0.009074 | Selected | Protective Factor |
| 5 | Abx | 0.034862 | Selected | Risk Factor |
| 6 | Probiotics | 1.533212 | Selected | Risk Factor |
| 7 | Vasopressors | 0.001089 | Selected | Risk Factor |
| 8 | Analgesics | -0.018724 | Selected | Protective Factor |
| 9 | Sedatives | 0.079732 | Selected | Risk Factor |
| 10 | K | 0.322886 | Selected | Risk Factor |
| 11 | Na | 0.024089 | Selected | Risk Factor |
| 12 | CRP | -0.001068 | Selected | Protective Factor |
| 13 | PCT | -0.010320 | Selected | Protective Factor |
| 14 | ENType | 0.095702 | Selected | Risk Factor |
| 15 | Rate | 0.004372 | Selected | Risk Factor |
| 16 | Temp | -0.176205 | Selected | Protective Factor |
| 17 | TubeType | 0.128745 | Selected | Risk Factor |
| 18 | Warmer | 0.194950 | Selected | Risk Factor |
| 19 | ENStart | -0.023177 | Selected | Protective Factor |
| 20 | DX | 0.000000 | Not Selected | No Effect |
| 21 | MVDays | 0.000000 | Not Selected | No Effect |
| 22 | RRT | 0.000000 | Not Selected | No Effect |
| 23 | Prokinetics | 0.000000 | Not Selected | No Effect |
| 24 | Alb | 0.000000 | Not Selected | No Effect |
| 25 | AdminMethod | 0.000000 | Not Selected | No Effect |

Optimal lambda value: 0.013299
